# Nursing Sensitive Outcomes evaluation in the Emergency Department: An Umbrella Review

**DOI:** 10.17533/udea.iee.v41n3e03

**Published:** 2023-10-21

**Authors:** Eartha Agatha Feller, Sofia Di Mario, Lucia Filomeno, Giuseppe La Torre

**Affiliations:** 1 Registered Nurse. Sapienza University of Rome, Italy. Email: eartha69@gmail.com Sapienza - Università di Roma Sapienza University of Rome Italy eartha69@gmail.com; 2 Registered Nurse, Ph.D student. Sapienza University of Rome, Italy. Email: sofia.dimario@uniroma1.it Sapienza - Università di Roma Sapienza University of Rome Italy sofia.dimario@uniroma1.it; 3 Registered Nurse, Ph.D student. Tor Vergata University of Rome, Italy. Email: lucia.filomeno@uniroma1.it. Corresponding author Università degli Studi di Roma Tor Vergata Tor Vergata University of Rome Italy lucia.filomeno@uniroma1.it; 4 Medical Doctor, Full Professor, Sapienza University of Rome, Italy. Email: Giuseppe.latorre@uniroma1.it Sapienza - Università di Roma Sapienza University of Rome Italy Giuseppe.latorre@uniroma1.it

**Keywords:** standardized nursing terminology, emergency nursing, nursing care, emergency service, hospital, terminología normalizada de enfermería, enfermería de urgencia, atención de enfermería, servicio de urgencia en hospital, terminologia padronizada em enfermagem, enfermagem em emergencia, cuidados de enfermagem, serviço hospitalar de emergência

## Abstract

**Objective.:**

The aim of this review was to identify reported nursing-sensitive outcomes in the Emergency Department to date.

**Methods.:**

An Umbrella review was conducted. Four databases, CINAHL, Pubmed, Web of Science and Scopus, were searched from inception until October 2022. MeSH terms were: "nursing", "sensitivity and specificity", "emergency service, hospital", "nursing care". Two reviewers independently screened studies against the inclusion criteria for eligibility, extracted data and assessed study quality with the SIGN tool. Results of the included studies were summarized and described in themes for narrative analysis. The study was enrolled in the PROSPERO registry (CRD42022376941) and PRISMA guidelines were followed.

**Results.:**

The search strategy yielded 2289 records. After duplicate removal, title, abstract and full-text eligibility screening, nine systematic reviews were included in the review. A total of 35 nursing-sensitive outcomes were reported. The most described outcomes were waiting times, patient satisfaction and time to treatment. The less measured were mortality, left without being seen and physical function. Synthesizing nursing-sensitive outcomes in themes for reporting, the most measured outcomes were within the safety domain (*n=20*), followed by the clinical (*n=9*), perceptual (*n=5*) and the least explored functional domain (*n=1*).

**Conclusion.:**

Nursing sensitive outcomes research in emergency nursing practice is a conceptual challenge still in its early stage. Several nursing-sensitive outcomes were identified in this review that can evaluate the contribution of emergency department nursing care to patient outcomes. Further research is required to explore patient outcomes sensitive to emergency nursing care.

## Resumen

**Objetivo.:**

Identificar los resultados sensibles de enfermería reportados en los Servicios de Urgencias.

**Métodos.:**

Se realizó una revisión general. Se hicieron búsquedas en cuatro bases de datos, CINAHL, Pubmed, Web of Science y Scopus, desde su inicio hasta octubre de 2022. Los términos MeSH empleados fueron: "nursing", "sensitivity and specificity", "emergency service, hospital", "nursing care". Dos revisores examinaron de forma independiente los estudios en función de los criterios de inclusión para determinar su elegibilidad, extrajeron los datos y evaluaron la calidad de los estudios con la herramienta SIGN. Los resultados de los estudios incluidos se resumieron y describieron en temas para el análisis narrativo. El estudio se inscribió en el registro PROSPERO (CRD42022376941) y se siguieron las directrices PRISMA.

**Resultados.:**

La estrategia de búsqueda produjo 2289 registros. Tras la eliminación de duplicados y el cribado de elegibilidad de título, resumen y texto completo, se incluyeron en la revisión nueve revisiones sistemáticas. Se informó de un total de 35 resultados sensibles a la enfermería. Los resultados más descritos fueron los: tiempos de espera, la satisfacción del paciente y el tiempo hasta el tratamiento. Los menos medidos fueron la mortalidad, el tiempo sin ser evaluado y la función física. Sintetizando los resultados sensibles a la enfermería en temas para la notificación, los resultados más medidos estaban dentro del dominio de la seguridad (*n*=20), seguidos por el clínico *(n*=9), el perceptivo (*n*=5) y el dominio funcional menos explorado (*n*=1).

**Conclusión.:**

En esta revisión se identificaron varios resultados sensibles a la enfermería que pueden evaluar la contribución de los cuidados de enfermería en los servicios de urgencias a los resultados de los pacientes. La investigación de resultados sensibles a la enfermería en la práctica de la enfermería de urgencias es un reto conceptual que aún se encuentra en su fase inicial.

## Resumo

**Objetivo.:**

Identificar resultados de enfermagem sensíveis notificados em Serviços de Emergência.

**Métodos.:**

Foi realizada uma revisão geral. Foram pesquisadas quatro bases de dados: CINAHL, Pubmed, Web of Science e Scopus, desde a sua criação até outubro de 2022. Os termos MeSH utilizados foram: "enfermagem", "sensibilidade e especificidade", "serviço de emergência, hospital", "cuidados de enfermagem". Dois revisores selecionaram independentemente os estudos em relação aos critérios de inclusão para determinar a elegibilidade, extraíram os dados e avaliaram a qualidade do estudo com a ferramenta SIGN. Os resultados dos estudos incluídos foram resumidos e descritos em temas para análise narrativa. O estudo foi registrado no registro PROSPERO (CRD42022376941) e as diretrizes PRISMA foram seguidas.

**Resultados.:**

A estratégia de busca produziu 2.289 registros. Após remoção das duplicatas e triagem do título, resumo e texto completo para elegibilidade, nove revisões sistemáticas foram incluídas neste estudo. Foram relatados 35 resultados de enfermagem sensíveis, sendo os mais descritos: tempo de espera, satisfação do paciente e tempo para tratamento. Os menos frequentes foram: mortalidade, tempo sem avaliação e função física. Sintetizando os resultados sensíveis à enfermagem por meio de tópicos de relato, os mais mensurados foram dentro do domínio segurança (*n*=20), seguido do domínio clínico (*n*=9), do perceptual (*n*=5) e do funcional. menos explorados (*n*=1).

**Conclusão.:**

Esta revisão identificou vários resultados sensíveis à enfermagem que podem avaliar a contribuição dos cuidados de enfermagem nos serviços de urgências para os resultados dos pacientes. A investigação de resultados sensíveis na prática de enfermagem em emergências é um desafio conceitual que ainda está em fase inicial.

## Introduction

Resource constraints driven health service reforms^1^^-^^4^ and strategies to improve safety and quality of patient care.^2^^,^^4^^,^^5^ These are a high priority for health care systems worldwide.^2^^,^^3^^,^^5^ The demand for professional,^1^^,^^4^^-^^6^ and budgetary^7^^,^^8^ accountability within healthcare, imposes nurses and nursing managers to provide evidence of nursing care quality^1^^,^^4^^,^^6^ and to implement appropriate strategies. Nurses embody the largest professional component in hospital settings^1^^,^^5^^,^^9^^,^^10^ and are present at all levels of the healthcare system.^1^^,^^5^^,^^7^^,^^9^ Nurses deliver most direct care to patients 24 hours a day,^1^^,^^7^^,^^9^, with their actions having a major impact on patients’ outcomes.^5^^,^^9^ As nurses also account for a considerable fraction of hospitals’ operating costs,^1^^-^^3^ it becomes mandatory to be able to measure and demonstrate their peculiar contribution to patient outcomes.^1^^,^^8^^,^^11^^,^^12^

Emergency Departments (EDs) are a unique,^13^ dynamic, nurse-driven and high-paced environment, with no control over patient volume or severity.^14^ Nurses are the first professionals to assess and start treatment according to guidelines for all patients entering the ED.^13^^-^^15^ In the last decades, increasing demands,^13^^,^^16^^,^^17^ the ageing population,^17^ overcrowding^13^ and boarding^16^ have put a strain on ED nurses. They are challenged daily in delivering life-saving patient-centered and evidence-based care, in a timely, safe, equal and effective manner.^12^^,^^16^ The extended scope of practice of ED nurses^13^^,^^15^^,^^16^ is to meet service demands,^13^ calls ED nurses’ awareness and accountability for provided care. Conversely, the effectiveness of nursing care on patient outcomes is still invisible to healthcare executives,^1^^,^^5^^,^^7^^,^^14^^,^^16^ patients, public opinion and other healthcare professionals.^18^ Lastly, nursing care impact is not represented in healthcare performance databases.^1^^,^^4^^,^^5^^,^^7^

Nursing Sensitive Outcomes (NSOs) or nursing sensitive indicators^4^^,^^8^^,^^11^ are metrics that reflect nursing care quality^6^^,^^10^^,^^14^ and express the contribution of nursing to patient outcomes.^2^^,^^6^^,^^11^ NSOs are the criteria for health status changes that can be directly^14^ or indirectly^8^ affected by nursing care. Therefore, NSOs are outcomes relevant and based upon nurses’ scope and domain of practice, where evidence has linked nursing inputs or interventions with patient outcomes.^(7,9,^^19^^, 20)^ Several countries developed national or regional nursing outcomes database registries,^1^^,^^4^^-^^6^^,^^8^^-^^10^^,^^12^^,^^19^ that focus on the impact of nursing care in hospital settings,^8^^,^^9^ to support evidence-based healthcare practice^4^^,^^5^ with Structure-Process-Outcome indicators.^5^^,^^8^ Thus, NSOs measurement can empower benchmark performance,^4^^-,6,9,12,19)^ evaluate and improve effectiveness of nursing interventions^4^^,^^7^^,^^9^ and can provide feedback about areas in need of improvement to nursing executives and policymakers ^(^^5^^,^^7^^,^^9^^,^^10^. NSOs have been identified in various acute care settings,^4^^,^^6^^,^^21^^,^^22^ but there is a lack of specific outcomes that express the wide scope of ED nursing care.^14^ Moreover, outcomes suitable in certain settings may not be appropriate for the ED context.^22^ The overall aim of this review, was to explore available evidence on NSOs research in the ED, to identify which patient outcomes sensitive to nursing care are reported in this setting. The review question for this study was: *“What nursing-sensitive outcomes can we assess in the Emergency Department?”* In response to the research question, an umbrella review was undertaken to summarise all evidence from multiple systematic reviews consistently. A review of systematic reviews enables a comprehensive understanding of existing research on NSOs measured in the ED to this point.

## Methods

Identification of relevant studies. Prior to starting the review, a research protocol was developed and the Prospero register was checked to determine whether similar reviews were already performed or underway. There were no studies exploring NSOs measuring in the ED at the time of consultation. A search strategy was designed. The research protocol for the current umbrella review was documented in the PROSPERO registry (CRD42022376941). The umbrella review was conducted in accordance with the Preferred Reporting Items for Systematic Reviews and Meta-Analyses (PRISMA) guidelines.^23^ CINAHL, PubMed, Scopus, and Web of Sciences databases were explored from inception until October 2022, to ensure all relevant studies were captured. Searching terms were based on elements identified in the research question, combining free text and Boolean terms. Searching terms were adapted for each database interface. Key search concepts were: *“nursing sensitive outcomes”, “emergency department” and “nursing care”*. Retrieval was limited to systematic reviews written in English or Italian concerning the ED adult population. 

**Figure 1:**
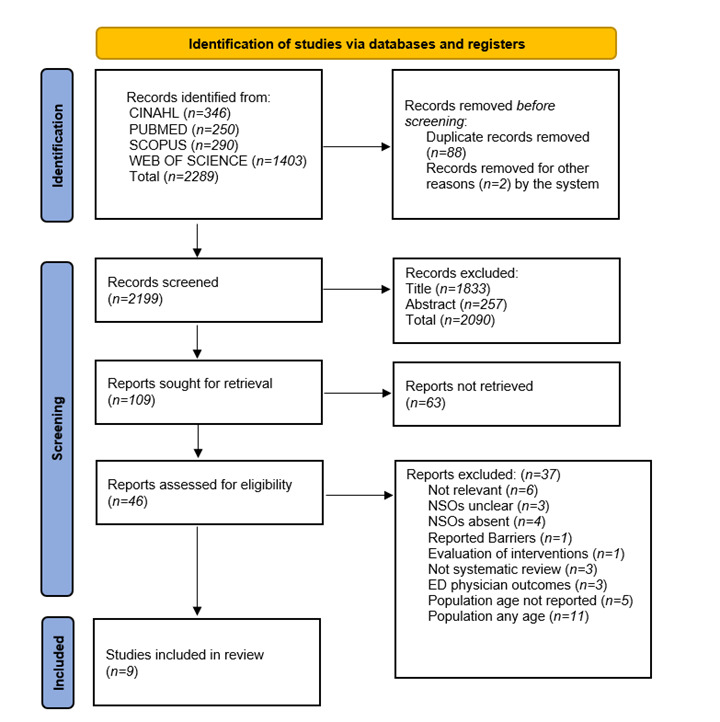
PRISMA Flow Chart study selection and screening process^23^

Study selection and Eligibility criteria. Systematic reviews were selected for inclusion, only when they met the following Population, Intervention or Exposure, Comparator, Outcome, Study design (PI[C]OS) criteria: (a) Population: adult patients (> 18 years) admitted to the ED receiving nursing care (b) Intervention: nursing care or interventions provided in the ED (c) Outcome: any evidence on the association between emergency nursing care and the evaluation of NSOs (d) Study design: studies with a systematic review design. Thus, papers were excluded when (a) concerning the paediatric population (< 18 years) (b) they were not relevant to the research question (c) focusing on settings other than the ED (d) and without a systematic research design. Search results were collected into the Zotero reference manager and duplicates were removed. Titles and abstracts were independently screened by two authors against the inclusion criteria. Two reviewers independently assessed the full text articles for eligibility against the PI(C)OS criteria and final review inclusion. Any disagreement, at each screening stage, was solved through consensus of a third reviewer. The comprehensive screening process is reported in the PRISMA flow chart in Figure 1. 

Protocol deviation. Due to the heterogeneity found in the selected studies when outlining the adult population criteria, the review inclusion criteria for the population (>18 years) was adopted. Any review reporting and stating adults as an inclusion criterion were included. In studies contemporarily investigating adult and paediatric populations, only adult data were considered for evaluation. 

Data extraction. Two authors independently conducted data extraction from each study using a pre-customized spreadsheet. Study characteristics included: First author and year, title, study design, rating of quality, objective, results and NSOs measured. Extracted data were summarised and synthesised for narrative and descriptive analysis. Disagreements among reviewers were resolved by consensus involving a third reviewer.

Quality Assessment. Methodological quality of the systematic reviews was assessed independently by two researchers using the SIGN Checklist for Systematic Reviews and Meta-Analyses developed by Healthcare Improvement Scotland.^24^ The SIGN tool consists of 12 questions for assessing study integrity and 2 questions for overall assessment. Each question is answered with the options yes or no, and when appropriate can’t say or not applicable. Two reviewers independently assessed the methodological quality of the included studies. A study quality score was calculated for each included study (low, moderate, or high quality), and was displayed in the extraction Table 1. Any discrepancies during the quality assessment were resolved by discussion and consensus by a third reviewer. 

## Results

The search strategy yielded 2289 records. After removing duplicates, titles and abstracts (*n=2199*) were screened. Full texts of 46 remaining reviews were assessed for eligibility. Finally, nine studies were included in the umbrella review. Included studies were published between 2007 and 2021. Full details and characteristics of the included studies are presented in Table 1. Studies originated from Ireland (*n=1*), Sweden (*n=1*), Canada (*n=1*), and Australia (*n=6*) being the most cited country. Study samples from systemic reviews (*n=167*) ranged from four^25^ to thirty six.^26^ All nine included studies were systematic reviews of which one performed meta-analysis of RCT,^25^ and two^27^^,^^28^ also performed meta-analysis for results. While four studies could not accomplish meta-analysis due to limited data^29^ or heterogeneity of studies.^30^^-^^32^ The reviews evaluated topics related to nurse-initiated interventions (*n=3*), triage (*n=2*), discharge management (*n=2*) and the impact of Nurse Practitioners (*n=2*). The nine included systematic reviews reported a total of 35 nursing-sensitive outcomes (Table 1). The most studied nurse-sensitive outcome was waiting time (*n=5*) followed by patient satisfaction (*n=4*), LOS (*n=3*), and time to analgesia (*n=2*). The least investigated outcomes, each reported in one study, were physical function, mortality and left without being seen (LWBS).

The nine systematic reviews underwent methodological quality assessment using the SIGN tool and were rated from high,^25^^,^^27^^,^^28^^,^^32^^,^^33^ acceptable^26^^,^^29^^,^^30^^,^^31^ to low.^32^ Comprehensive literature search was performed by all reviews. One study had a registered protocol prior to beginning the review.^31^ Several studies (*n=4*) needed to be clarified about the selection of studies in duplicate, while one acknowledged this shortage.^32^ All studies conducted data extraction with two authors and the characteristics of included studies were outlined in a table. One study did not list the reasons for study exclusions.^33^ All included reviews used a wide variety of study quality assessment tools. Two reviews declared receiving partly resource funding for the research.^25^^,^^31^

**Table 1:** Description of the included studies.

N°	Authors and year	Title	Study Design	SIGN Quality Rating	Objective	Results	Nursing Sensitive Outcomes (NSOs)
1	Burgess *et al.*, 2021	The effectiveness of nurse-initiated interventions in the Emergency Department: a systematic review	Systematic Review	High	To determinate the effectiveness of nurse-initiated interventions on patient outcomes in the Emergency Department	Twenty-six studies were included, nine RTC and seventeen quasi-experimental designs. Nurse interventions may facilitate progression of care in the emergency department and have the potential to improve time-to-treatments and decrease hospital admission rates.	Time-to-treatment Pain level score Symptom relief Inpatient admission
2	Caliban *et al.*, 2017	A systematic review of the impact of nurse-initiated medications in the emergency department	Systematic Review	High	To evaluate the effects of nurse-initiated medications (NIM) in the emergency department and to quantify the impact of the practice on quality care indicators.	Five experimental studies were included. Nurse medications are safe and beneficial for emergency department patients.	Safety Timeliness Effectiveness Equitability Patient-centered care Efficiency
3	Carter *et al.*, 2007	A systematic review of the impact of nurse practitioners on cost, quality of care, satisfaction and wait times in the emergency	Systematic Review	Low	To evaluate the emergency setting, by looking specifically at four keys outcomes measures: wait time, patient satisfaction, quality of care and cost-effectiveness.	36 articles were included. The results of this review suggest that the addition of a staff member dedicated to seeing minor treatment patients will improve wait times for these patients as well as improve patient satisfaction, with little or no impact on quality care.	Cost Quality Satisfaction Wait time
4	Corkery *et al.*, 2021	What is the impact of team of triage as an intervention on waiting times in an adult emergency department? A systematic review	Systematic Review	Acceptable	To identify the impact of Team Triage (TT) on waiting time (WT) in adult emergency departments	12 studies were covered. four RCTs, four cohort studies and four quasi-experimental. Waiting times are improved with team triage and can enhance patient satisfaction, LWBS and mortality rates.	Waiting times
5	Dermody *et al.*, 2020	The effectiveness of pictorial discharge advice versus standard advice following discharge from the ED: a systematic review and meta-analysis	Systematic Review and Meta-Analysis	High	To determinate the effectiveness of pictorial discharge advice compared with standard discharge advice in the emergency department.	Four studies were included. This review supports the use of pictorial discharge advice, especially for increased comprehension and compliance with discharge advice.	Comprehension Compliance Patient satisfaction ED reattendance
6	Elliot *et al.*, 2021	Interventions for the discharge of older people to their home from the emergency department: a systematic review	Systematic Review	Acceptable	To evaluate the effectiveness of discharge interventions used for older people from the emergency department (ED) to their homes in the community by emergency clinicians.	Twenty-five studies met the inclusion criteria, thirteen RCTs and twelve quasi-experimental. Discharge interventions from the ED for older people are harmless and can be useful, but their effectiveness has yet to be proven in RCT studies.	Mortality ED representation after the index visit Physical function
7	Jennings *et al.*, 2015	The impact of nurse practitioner services on cost, quality of care, satisfaction and waiting times in the emergency department: a systematic review	Systematic Review	Acceptable	To establish the impact of nurse practitioner services on cost, quality of care, satisfaction and waiting times in the emergency department for adult patients.	Fourteen studies were covered, two systematic reviews, two quasi-RCTs and ten observational descriptive design studies. Emergency nurse practitioner services have a positive effect on quality of care, patient satisfaction and waiting times in the emergency department. Evidence on outcomes of cost-benefit analysis needs to be more comprehensive.	Patient satisfaction Waiting times for care Quality of care Costs
8	Oredsson *et a.l*, 2011	A systematic review of triage-related interventions to improve patient flow in emergency departments	Systematic Review	High	To identify and assess evidence of interventions improving patient flow in emergency departments .	Thirty-three articles were selected, notably RCTs with a control group or in observational studies with historical controls. Fast track reduces LOS and LWBS. Team triage can reduce LOS and LWBS. Limited evidence on the impact of nurse-requested X-rays on patient flow.	Waiting time (for physician assessment) Length of stay (LOS) Left without being seen (LBWS)
9	Varndell *et al.*, 2018	Quality and impact of nurse-initiated analgesia in the emergency department: A systematic review	Systematic Review	High	To examine the quality and impact of nurse-initiated analgesia (NIA) in adult patients presenting with acute pain in the ED.	Twelve studies were included, nine non-experimental and three quasi-experimental design studies. NIA protocols increase the likelihood to receive analgesia, in a safe and timely manner	Time to analgesia Waiting times ED length of stay Change in pain score Patient satisfaction Adverse events.

### Reporting NSO results

Findings of the identified NSOs were rationalized for narrative reporting in domains adapting the format used by Danielis et al.^21^ NSOs were categorized in four domains (Safety, Clinical, Functional and Perspective) following the Doran outcome classification^34^ and based on similarities. The most investigated sequential domains were safety (*n=20*), clinical (*n=9*), and perceptual (*n=5*). The least explored was the functional domain (*n=1*). 

### Clinical domain

Four studies^25^^,^^27^^,^^28^ ^32^ examined the clinical domain, which involves outcomes related to symptom control,^34^, goal assessment and monitoring of change in health status concerning patient’s illness and recovery in the ED.^21^ Pain was the most investigated outcome in studies (*n=3*) and was associated with nurse-initiated interventions. Pain levels were commonly measured using the 11-point numerical rating scale (NRS) or 0-100 mm Visual Assessment Scale (VAS) score and assessed at pre- and post-analgesia administration. Change in pain score or pain relief was described in one review^32^ as a > 50% decrease of initial pain level score or percentages of patients with ≥3-point reduction in pain score, within one hour after first analgesic delivery; decrease of > 33% in patient pain score while staying in the ED or < 4 on a 0-10 scale at discharge; a pain level reduction of > 2 points or more and up to < 4 points on a 0-10 scale; effectiveness, described in one review,^28^ was accomplished when adequate pain relief, with a 2 point reduction or more to initial pain score, and too mild intensity (<4) was reached at patients discharge. Equitability was established when patients presenting with moderate to severe pain were more liable to receive nurse-initiated analgesia when nurses were allowed to apply this intervention.^28^ Symptom relief, was reported in one study^27^ and defined as control, resolution or clinical assessment of the symptom using nurse-initiated interventions. One study evaluated outcomes with pictorial discharge instructions compared to standard discharge advice.^25^ Compliance was documented using the proportion of daily adherence to wound care instructions. While comprehension of discharge instructions was measured using a four-item questionnaire and discharge advice instructions readability. Quality appraisal of the reviews examining this domain was high.^25^^,^^27^^,^^28^^,^^32^ Pooled meta-analysis performed in three studies showed overall poor heterogeneity.^25^^,^^27^^,^^28^ Although one review reported removing one study for influencing heterogeneity and repeated analysis.^25^

### Safety domain

This domain relates to unintentional situations linked to the process of care that can lead to undesirable patient outcomes.^21^ All of them investigated safety-related outcomes, and these included waiting times (*n=5*), LOS (*n=3*), quality of care (*n=2*), costs (*n=2*), timeliness (*n=1*), time to analgesia (*n=1*), time to treatment (*n=1*), ED reattendance (*n=1*), inpatient admission (*n=1*), ED representation after index visit (*n=1*), safety (*n=1*), adverse events (*n=1*), mortality (*n=1*) and LWBS (*n=1*). Waiting times, were the most reported outcome^26^^,^^29^^,^^30^^,^^32^^,^^33^ and were measured as the intervening time between ED entrance and physician assessment,^33^ using team triage (triage nurse and physician),^30^ Rapid Assessment Team (RAT) and fast track streaming processes.^33^ Moreover, waiting times were explored in one study using the availability of NIA and the proportion of trained emergency nurses in NIA.^32^ Two studies reported wait times in association with the introduction of Nurse Practitioners in the ED, using the UK SEE and Treat model^26^ and collaborative models of care (NP and Resident physician) for throughput of ED patients.^29^ ED length of stay, the total time spent in the ED,^33^ was studied in three reviews^28^^,^^32^^,^^33^ using the efficiency of NIA^28^^,^^31^ or Nurse Initiated patients, the effect of ED point-of-care laboratory testing and the number of x-rays requested by nurses.^33^ The reviews addressing this outcome reported uniform evidence and were ranked high in methodological quality. 

Time to analgesia, delivering timely care, depends on the proportion of ED nurses educated in NIA, the availability of analgesia at the time of entrance to ED, and the implementation of NIA protocols or policies.^32^ Timelessness^28^ or time to analgesia, decreasing waits and unsafe delays at times for both the provider and receiver of care, was reported in one study and measured from arrival time in triage to first analgesic; heterogeneity of findings was significant and need to be inferred with caution. 

Time to treatment^27^ was investigated in one study and measured in minutes or hours; meta-analysis was not performed as variations in treatment protocols and analgesic type were high.^27^ Inpatient admission was evaluated using patient admission rates as a result of treatment nurse-initiated.^27^ ED Reattendance^25^ within 28 days was included in one review but was not measured by the included studies. Moreover, ED Representations after index visit^31^ in elderly patients receiving personalized health assessments and ED discharge interventions were documented using the proportion of ED representations within various time points of the ED index visit.

Safety-related to NIM was documented by two reviews^28^^,^^32^ using the occurrence of adverse events described as reduced consciousness level (GCS < 14), hypoxia < 90-92%, bradypnea < 10-12 b/min, bradycardia < 50-60 b/min, systolic BP < 100 mmHg, or episodes of vomiting and nausea. LWBS, the percentage of ED patients leaving without being seen by a physician, was investigated only in one study^33^ using the effect of a triage liaison physician supporting the triage nurse, evaluating ambulance patients, starting diagnostic procedures, and managing administrative issues. Lastly, mortality was reported in one review^31^ using various time points of evaluation. Quality of care was reported in two studies ^(^^26^^,^^29^ associated with Emergency NP services effectiveness and was measured using adverse events and health status follow-up as a combination score from patient satisfaction. Other measures used to define quality of care were unsuitable management of patients, x-ray accuracy interpretation, LWBS, unforeseen or unplanned returns of patients to the ED and rates of missed injuries. Costs as un outcome were measured in two studies,^26^^,^^29^ and evaluated NPs’ capacity to ration recourses by using the management of patients with soft tissue injury and the compliance to clinical decision guidelines (e.g., Ottawa ankle rule, follow-up scheduling) compared with residents.

### Functional domain

The functional domain, which is recognized as patients’ independence in activities of daily living, physical abilities and psychosocial functioning,^21^^,^^34^ was investigated only in one review.^31^ This systematic review^31^ explored the effectiveness of personalized discharge health interventions for elderly ED patients in their homes. Physical function was measured using the Function Measurement Tool [FMT], Older American Resources and Services Scale [OARRS] and the Modified Barthel Index-50 (MBI) score. The methodological quality of the study addressing this outcome was ranked by the SIGN tool as acceptable. 

### Perspective domain

Five studies^25^^,^^26^^,^^28^^,^^29^^,^^32^ evaluated the perspective domain, which investigates the experience of the patient with nursing care received in the ED, and embraces the outcomes produced by the ED environment.^21^^,^^34^ The most investigated outcome was patient satisfaction (*n=4*) followed by patient-centeredness (*n=1*). Patient satisfaction with NP fast-track services and ED care delivery compared to resident physicians was investigated.^26^^,^^29^ Patient satisfaction was measured using an adapted 11-item Strategic and Clinical Quality Indicators within the Postoperative Pain Management questionnaire; a rating scale (1-10) with a single question to measure patient’s satisfaction with NIA during pre- and post-implementation; a six questions patient satisfaction questionnaire. One study ^(^^25^ reported patient satisfaction with discharge advice and was defined as the proportion of patient reporting “very satisfied”. Patients centeredness in nurse-initiated medications was documented in one study ^(^^28^ and was assessed as patient satisfaction, using a 10-item questionnaire with a 5-point Likert scale for each item. The quality of studies representing this domain where either acceptable^26^^,^^29^ or high.^25^^,^^28^^,^^32^

## Discussion

The primary aim of this umbrella review was to assess how patient outcomes sensitive to nursing practice have been monitored in ED up to the present. This resulted in 35 nursing-sensitive outcomes, representing nine systematic reviews published over 2007-2021, that could reflect the quality and safety of nursing care for the ED adult population. Although the search strategy yield several studies (*n=2289*), the small sample of included studies (*n=9*) could be deficient in representing the comprehensive universe of potential ED nursing-sensitive outcomes and nursing practice.^14^ This may suggest that NSOs’ research for the emergency department setting is still germinal.^1^ Furthermore, the quality of evidence was variable (low, acceptable or high). The majority of the studies were conducted in Australia (*n=6*). Hence, when interpreting findings it is essential to take into account the study’s geographical area of origin^26^ as EDs worldwide may differ in healthcare system, logistics, organizational standards, models of care and ED nursing roles. Several of the included reviews documented the impact of nurse-initiated interventions (*n=3*) and the nurse practitioner role (*n=2*), and their contribution to patients’ outcomes. This may illustrate the prevalence of emergency nurses’ dependent and interdependent roles^21^^,^^34^ in current EDs as a result of the extended role and changes in the scope of practice of ED nurses^13^^,^^15^ in the last two decades.

Outcomes included in the safety domain were the most explored and involve aspects linked to the process of care that can lead to unintentional, undesirable patient outcomes. The focus on safety measures is understood within the intrinsic goals of nursing practice^11^ as nurses are accountable for keeping patients safe.^11^^,^^13^^,^^34^ Patient safety is recognized as an important indicator of nursing care with the purpose to prevent errors and adverse events, identifying and reducing the occurrence of potential harm.^1^^,^^11^ Moreover, research studies often select safety outcomes since data is ready to assess (e.g. hospital administrative data, discharge charts)^34^ and in an effort to determine best practices in the ED that can warrant safety for patients.^22^ The time-related factor (e.g., waiting times for treatment, care, analgesia or physician and overall time spent in the ED) was the most investigated outcome, a typical and critical ED performance indicator of care effectiveness;^29^ prolonged waiting times, can evolve in additional negative outcomes such as mortality, LOS and adverse events.^29^

Pain was the most investigated outcome included in the clinical domain. Pain outcomes are nurse-driven and employ NI protocols.^27^^,^^28^^,^^32^ Though, improved outcomes in analgesia rates using NIA are reported, results may depend on local settings^27^ and contributing factors may be demanding to establish.^32^

In the perspective domain patient-centeredness, which was synonymously to patient satisfaction,^28^ was linked to nursing interventions such as NP fast track compared to resident physicians, NIA protocols, and nurse-initiated medications, and discharge advice. This tendency supports the good levels of patient satisfaction outcomes with emergency nurse practitioner services^26^ compared to resident physicians^29^ and seems to be associated with nurse-initiated analgesia.^28^^,^^32^ However, methods evaluating patient satisfaction either failed in appropriate description or showed paucity.^32^ Warranting the value of patients’ EDs experience must be underlined since patients are key stakeholders in healthcare.^29^ Patients’ satisfaction with care depends on various aspects and can be affected by overall ED care experience, perception of quality of care, communication with staff and expediency of treatment, which makes measurement challenging.^28^ Therefore, validated patient satisfaction tools are needed for NSOs evaluation.^32^

The functional domain was the less explored for NSOs in the ED. The physical function was the only outcome reported in this domain by one study,^31^ exploring the effectiveness of discharge interventions for elderly ED patients. While metrics measuring this outcome (*n=3*) were substantial and methodological quality was acceptable the study sample is too small to acknowledge evidence. However, investigating outcomes of discharge processes, especially for populations at high risk (e.g. the elderly person, chronic or end-of-stage renal disease patients, deviant vital signs at discharge, and citizens with social medical insurance), is important to reduce return visits in the ED and to prevent adverse patient outcomes.^16^^,^^17^ Thus, therefore investigating positive outcomes measures to a greater extend, such as the functional status, may better demonstrate the effectiveness of ED nurses’ contribution.^6^^,^^7^ Gaining data that measures the functional status can be demanding and this may explain poor research.^6^^,^^7^

Limitations. This umbrella review has several limitations. The overall process of screening and selecting studies together with categorizing the outcomes for reporting and synthesizing findings was a challenge: Firstly, the differences between reviews in the definition of the adult population criteria has resulted in an adaption of the inclusion criteria of this review. Likewise, limitations in the population criteria may have resulted in the exclusion of studies that otherwise may have been eligible. The majority of the included studies were conducted in Australia therefore may present culture bias when interpreting the results. The selection, inclusion and extraction of data in studies were demanding owing to indistinct definitions and descriptions of outcomes (e.g. wait times, change in pain score, pain relief, quality of care); variations in conceptual framework used (e.g. self-constructed, quality dimensions of healthcare, clinical themes); variations in methods used to measure outcomes (e.g. quality of care, mortality). Lastly, outcomes were clustered in domains combining an intuitive approach, with adapted methods performed in studies^21^^,^^34^ which may create bias. 

Conclusion. The aim of this umbrella review was to outline the nursing-sensitive outcomes that have been evaluated in literature to date for the emergency department. In this review, 35 nursing-sensitive outcomes were identified across 9 studies, which could be relevant to the evaluation of the contribution of ED nursing care to patient outcomes. Findings showed that ED nursing-sensitive outcomes regarding the functional domain (e.g. physical function) were less investigated, while safety, clinical and perspective domains were more explored. NSOs research in emergency nursing practice is a conceptual challenge still in its early stage. Therefore, a standardized language is warranted within nursing to guide the development, classification, utilization and benchmarking of NSOs in the ED. Further research is needed to explore NSOs that makes the contribution of ED nursing practice visible.

NSOs research in emergency nursing practice is a conceptual challenge still in its early stage. Several nursing-sensitive outcomes were identified in this review that can evaluate the contribution of ED nursing care to patient outcomes. However, professional consensus is needed for agreed definitions and categorization of outcomes, formal methods, a conceptual framework and validated tools, to support the evaluation of nursing-sensitive outcomes and improve the quality of nursing care for patients admitted in the ED. Further research is required to explore patient outcomes sensitive to emergency nursing care to reflect the contribution of ED nursing practice. 

Implications for the practice. The NSOs identified in this review could be used to create an ED minimum dataset^14^^,^^18^^,^^21^ to outset the foundation for NSOs research in the ED. And, therefore, make the impact of nursing interventions on patient outcomes measurable, improvable and visible to stakeholders. A standardized language could guide ED nurses and managers to shift ED nursing practice to an outcome-based culture of quality ED nursing care. (12,14,19) Research in ED NSOs should focus not only on negative outcomes (e.g. adverse events, complications, safety) typical for high pasted environments, but also on positive outcomes (e.g. functional status, patient satisfaction, aspects of the clinical domain). Furthermore, research development for NSOs within the functional domain for ED care transition interventions (e.g. discharge, handovers) in an ever ageing ED population are needed. Lastly, the extended scope of practice and the uptake of ED nurse-initiated interventions requires validated and common tools to measure their effectiveness.
